# Experiences Among Patients With Cystic Fibrosis in the MucoExocet Study of Using Connected Devices for the Management of Pulmonary Exacerbations: Grounded Theory Qualitative Research

**DOI:** 10.2196/38064

**Published:** 2024-01-23

**Authors:** Maxime Morsa, Amélie Perrin, Valérie David, Gilles Rault, Enora Le Roux, Corinne Alberti, Rémi Gagnayre, Dominique Pougheon Bertrand

**Affiliations:** 1 Adaptation, Resilience and Change Research Unit University of Liège Liège Belgium; 2 Laboratory Health Promotion and Education (UR3412) Sorbonne Paris North University Bobigny France; 3 Paediatrics CF Centre Nantes University Hospital Nantes France; 4 Laboratoire Educations et Pormotion de la santé Université Sorbonne Paris Nord Bobigny France; 5 Institut National de la Santé et de la Recherche Médicale (UMR 1123 ECEVE) Université de Paris Paris France; 6 Unit of Clinical Epidemiology (CIC-EC 1426) Hôpital Universitaire Robert Debré, Assistance Publique des Hôpitaux de Paris Institut National de la Santé et de la Recherche Médicale Paris France

**Keywords:** cystic fibrosis, mobile health, mHealth, patient education, chronic disease, empowerment, devices, patients, detection, treatment, respiratory, education, monitoring, care

## Abstract

**Background:**

Early detection of pulmonary exacerbations (PEx) in patients with cystic fibrosis is important to quickly trigger treatment and reduce respiratory damage. An intervention was designed in the frame of the MucoExocet research study providing patients with cystic fibrosis with connected devices and educating them to detect and react to their early signs of PEx.

**Objective:**

This study aims to identify the contributions and conditions of home monitoring in relation to their care teams from the users’ point of view to detect PEx early and treat it. This study focused on the patients’ experiences as the first and main users of home monitoring.

**Methods:**

A qualitative study was conducted to explore patients’ and professionals’ experiences with the intervention. We interviewed patients who completed the 2-year study using semistructured guides and conducted focus groups with the care teams. All the interviews were recorded and transcribed verbatim. Their educational material was collected. A grounded analysis was conducted by 2 researchers.

**Results:**

A total of 20 patients completed the study. Three main categories emerged from the patients’ verbatim transcripts and were also found in those of the professionals: (1) *task technology fit*, reflecting reliability, ease of use, accuracy of data, and support of the technology; (2) *patient empowerment through technology*, grouping patients’ learnings, validation of their perception of exacerbation, assessment of treatment efficacy, awareness of healthy behaviors, and ability to react to PEx signs in relation to their care team; (3) *use*, reflecting a continuous or intermittent use, the perceived usefulness balanced with cumbersome measurements, routinization and personalization of the measurement process, and the way data are shared with the care team. Furthermore, 3 relationships were highlighted between the categories that reflect the necessary conditions for patient empowerment through the use of technology.

**Conclusions:**

We discuss a theorization of the process of patient empowerment through the use of connected devices and call for further research to verify or amend it in the context of other technologies, illnesses, and care organizations.

**Trial Registration:**

ClinicalTrials.gov NCT03304028; https://clinicaltrials.gov/ct2/show/results/NCT03304028

## Introduction

### Background

Pulmonary exacerbations (PEx) are the main cause of decline in lung function in patients living with cystic fibrosis (CF), representing the leading cause of death. Recommendations emphasize the importance of diagnosing PEx early to treat patients effectively and for them to have the best chance of regaining their previous baseline lung function after treatment [1]. Identifying warning signs of PEx requires many skills from patients daily—studies have shown that they must be able to monitor a combination of physiological parameters and patient-reported perceptions, such as weight loss, decreased spirometry, increased coughing, or increased sputum production reported daily, to diagnose PEx episodes and put in place the appropriate treatment [2,3]. Nevertheless, patients living with CF do not systematically monitor these warning signs, as few are equipped with devices to monitor variations in their physiological parameters or their perceptions over time, with the exception of patients who have received a lung transplant, who may be equipped with spirometers to detect a decrease in their respiratory function, which is a warning sign of acute rejection. However, patients need to access accurate and reliable measurements to monitor their lung function.

In recent years, a contemporary trend has emerged in health improvement and disease prevention: the “quantified self.” It refers to the quantitative measurement of various parameters linked to the state of one’s health (eg, heart rate and weight) or to lifestyle (eg, diet and physical activity) to monitor a disease or improve well-being. The premise is that one cannot improve what they cannot quantify. This quantification, which was still difficult to achieve a few years ago, has become more accessible through the development of new technologies and connected devices. These devices are connected to the internet and can collect, store, process, and transmit health-related data through sensors [4].

Connected devices can help patients gain a better understanding of disease and treatment and increase their levels of satisfaction and adherence to treatment when combined with patient education interventions [5,6]. Patient education is an empowerment approach for patients with chronic diseases aiming to improve their understanding and adherence to treatment by transferring knowledge from health care providers to patients through educational workshops and also by using patients’ experiential knowledge, which helps them adjust their management of the disease in their daily lives [7]. Patient education is known to have a significant positive impact on bioclinical indicators and on the well-being of patients [8]. Connected devices would act as a learning aid for patients by promoting real-life behavioral experimentation thanks to quick (or even immediate) access to objective data and to the development of knowledge about oneself anchored in one’s memory [9]. The use of connected devices by patients in their daily lives allows them to transfer what they learned during the workshops provided by health care providers to real-life situations, thus expanding on patient education. Experiential and continuous learning is facilitated when it is supported by health care providers to learn to interpret real-life data and compare them with the data collected at the hospital.

This way, connected devices could promote the process of empowerment, a concept that is understood as the development of patients’ ability to identify and meet their own needs, solve their own problems, mobilize the necessary resources to take action, and feel that they are in control of their health and their own lives [10]. According to Funnell and Anderson [11], empowerment is a process that is facilitated by counseling, educational, or psychological techniques to help the individual take control of the day-to-day management of their illness.

Currently, data are scarce on how connected devices are used in real-life situations by patients with chronic diseases and on how they influence knowledge of oneself and of one’s body, health, and disease [12]. However, we know that the dropout rate of connected devices can be high because of how cumbersome their use may be or the fact that they are too pressing a reminder of the person’s disease in their daily life [13,14], whereas adherence is mainly observed in young people and high-income socioprofessional categories who are more familiar with new technologies [15]. People’s experiences of using such connected devices vary depending on the person, the context, and their care environment. Therefore, the assessment of health technology is now moving toward a contextualized, patient-based evidence approach. According to this approach, the evaluation of eHealth devices is based on knowledge that originates directly from patients about their experiences of health, quality of life, and health services [16]. This approach is represented internationally by the work of the Warwick Patient Experiences Framework or the National Institute for Health and Care Excellence Patient Experience Guideline Development Group [17].

Drawing from the humanities and social sciences, it is now recommended for qualitative studies to be centered on patients’ feedback to understand the processes through which connected devices facilitate their acquisition of knowledge (of the body, risks, and diseases), in particular through the intimate and empirical experiences of the quantified body translated into data [18].

### Objectives

Therefore, we conducted a qualitative study with patients living with CF and with specialized CF centers in metropolitan France to explore the processes through which connected devices become an essential part of patients’ knowledge to allow them to self-manage their health and to contribute to a theory of individual patient empowerment through technology. The aim of the study was to understand how patients and health care providers lived and perceived this new intervention based on connected devices associated with patient education workshops to identify the contributions and conditions of home monitoring. The work is focused on stakeholders’ experience with the intervention. This study is part of an interventional project based on the hypothesis that an intervention that combines the provision of connected devices set up with personalized alert thresholds and a patient education intervention by health care providers can enable patients with CF to detect early signs of PEx and begin managing it themselves in a timely manner. For this self-management process to lead to the implementation of appropriate patient behavior, it is assumed that the educational intervention teaches patients to identify and respond appropriately to alerts.

## Methods

### Overview

The MucoExocet (from the French for “Cystic Fibrosis Exacerbation Connected Devices Therapeutic Education”) study, a pilot interventional study, was conducted from 2018 to 2021 and involved 22 adults and 14 adolescents (aged >12 years) with CF to assess whether the use of connected devices was feasible and useful to detect and treat PEx early (trial registration: ClinicalTrials.gov NCT03304028). As part of the overall research project, this qualitative study explored the users’ experiences at the end of the intervention. The intervention and protocol have been extensively described previously [19]. We used the EQUATOR (Enhancing the Quality and Transparency of Health Research) standards for reporting qualitative research elaborated by O’Brien et al [20] to present our study design.

### Summary of the Intervention in Its Context

Since 2005, a national organization associating health care providers from CF centers and patients and parents in France has been working to define the patient and parent competency framework (in pediatrics) and the associated set of educational tools. A therapeutic education tool named “React to PEx” (“Réagir en cas d’exacerbation”) was used to support patients’ and parents’ self-management of PEx episodes at home (Multimedia Appendix 1).

**Figure 1:**
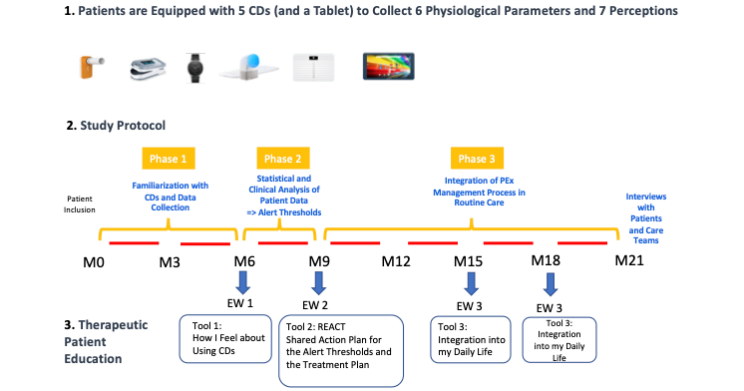
Design of the study intervention. CD: connected device; EW: educational workshop; M0: month 0; M3: month 3; M6: month 6; M9: month 9; M12: month 12; M15: month 15; M18: month 18; M21: month 21; PEx: pulmonary exacerbation.

The intervention designed for the MucoExocet study combined the provision of connected devices with an educational program based on the React to PEx tool. It was renamed “React with CDs” and incorporates measurements from connected devices and personalized alerts (Figure 1). The goal of the intervention was to develop the patients’ (or parents’) ability to take action at the first signs of exacerbation identified through measurement deviations by connected devices. For this study, connected devices were used to collect 13 parameters, including 6 physiological parameters measured by the devices (forced expiratory volume in 1 second [FEV1], cardiac frequency, arterial hemoglobin oxygen saturation, weight, sleep duration [min/night], and physical activity [step count/d]) and 7 patient-reported perceptions described using emoticons in a journal provided by the spirometer application (trouble breathing, need for more airway clearance, increased symptoms at night, difficulty performing usual activities, greater fatigue, loss of appetite, and change in sputum [color or quantity]). At the request of both physicians and patients, the option chosen in the study was to not send the data collected via connected devices to the physician but only to the patient. However, the data could be shared during a consultation at the center or during a phone call or email exchange if the patient (or parent) wished to do so.

Thresholds of irregular variation for patients’ parameters were calculated using the cumulative sum control chart method based on the data collected during phase 1 of the implementation of connected devices (3 months); this allowed physicians to set alert thresholds for each parameter and each patient during the first educational workshop with patients. Thus, alerts could be sent by email to the patients or parents throughout the period of routine monitoring using connected devices (12 months). An action plan was agreed upon by the physician and the patient or parent during this educational workshop to respond to alerts.

### Development of the Educational Tool and Educational Intervention in the Centers

One of the centers played a leading role in the implementation of the study because of its leading role in therapeutic education for patients with CF in France (the CF center in Nantes). During phase 2, the physician and therapeutic patient education nurse developed the different educational tools for the 3 stages of the program (educational workshops 1-3) based on the statistical analyses of patient data and with the participation of an adult patient expert and tested these tools with a parent and an adolescent patient from their center. The tools and educational program are described in the publication cited previously [19].

Beyond the patient recruitment process, a physician and a health care provider (nurse or physiotherapist) from each investigating center were involved in handing the connected devices to the patients or parents, setting them up with the patients or parents and explaining how to use them, solving technical problems with the patients or parents with the help of the device suppliers, and participating in the interpretation of statistical analysis of the data collected during the implementation phase (3 months) to define personalized alert thresholds for their patients. They were trained in the use of the React with CDs educational tool to agree with their patients on an action plan in the event of PEx; they educated their patients in the 3 sessions provided as part of the educational program (“Your impressions of using connected devices during the implementation phase” and “Your action plan for responding to exacerbation warning signs, and Review of your action plan after 6 months of routine monitoring”). At the end of the study, health care providers participated in a focus group to report on their experience with the study and this monitoring method.

### Study Population and Study Centers

The centers were selected by the research group on a voluntary basis among centers who had participated in the quality improvement program. They were familiar with the educational tool “React to an exacerbation.” The 7 CF centers were selected to include patients and their families of various conditions of life, economic statuses, and geographic areas (either urban or rural). Finally, the 7 centers were located in 4 different geographical areas; 3 (43%) were pediatric centers (4 patients per center), and 4 (57%) were adult centers (6 patients per center). None had previous experience with connected devices for their patients at the initiation of the study. In total, 36 patients, adults or adolescents, were included in the MucoExocet study. The sample was defined according to the recruitment capacities of the centers and the possibility of observing a saturation phenomenon in the qualitative study [21].

The recruitment process conducted by physicians in the centers was carried out based on patients’ voluntary participation and their interest in using new technologies. The participant inclusion criteria were as follows: age of ≥12 years, clinically stable condition (no PEx requiring intravenous antibiotics within the previous 4 weeks), at least one PEx within the previous 12 months, current follow-up at a participating CF center (and no plans to change centers during the course of the study), no history of having undergone solid organ transplants, prescription of at least one pulmonary medication (eg, inhaled mucolytic, inhaled or oral antibiotic therapy, or hypertonic saline), ability to speak French, ability to connect a tablet to Wi-Fi, and provision of written informed consent.

The number of individuals to be recruited took into account an estimated dropout rate of 20%. A form was offered to the patients leaving the study to identify the main reasons for their withdrawal (Multimedia Appendix 2).

### Data Collected by the Patients Using the Connected Devices

The 13 parameters were collected using 5 different connected devices, and a few of them collected more than one parameter. During the first 3 months, data were to be collected twice a week. During the routine phase, the frequency was agreed upon between the patient and the care team from twice a week to once every 2 weeks depending on the patient’s health outcomes and life conditions. The procedure used to collect the data was explained in a document given to the patients at inclusion (Textbox 1).

Owing to the great variety of measurements taken, the time spent on the measurements was not recorded.

**Procedure to collect the data.**

- Data collected without any participation from the patient (sleep, steps, and cardiac frequency): the duration of sleep was measured by the sensor under the mattress, and the step count and cardiac frequency were measured by the watch.
- Clinical data (spirometry and oxygen) required patient participation; at the end of the spirometry measurement, emoticons were presented for each of the 7 perceptions.
- Weight was expected to be measured in the morning (naked) the same day as the clinical data.

### Qualitative Data Collection

Patients’ experiences were collected through semistructured interviews using an interview guide with 8 open-ended questions (Textbox 2), derived and adapted from validated protocols for patient narrative elicitation in outpatient care experiences [22]. The experience and workload of the care teams were explored in focus groups using an interview guide with 5 open-ended questions (Textbox 3).

Three sources of data were collected: (1) data collected during patient or parent interviews using an interview guide with open-ended questions (Textbox 2), (2) data regarding the educational program with the physician (the documents completed by the patient and the clinician, including the personalized action plan in case of PEx), and (3) data from the focus groups with care teams at the end of the study using a semistructured guide (Textbox 3).

**Guide for the semistructured interviews with patients or parents.**

1. For you, what are the most important aspects in the management of your respiratory exacerbations in your daily life?
2. How do you rate the conditions for managing exacerbations during the study (based on what is most important to you)?
3. Can you tell us about a positive experience you had during this study concerning the management of your exacerbations? What happened and how did it make you feel? Did you do anything in particular after this positive experience (eg, change your attitude or behavior)?
4. Can you tell us about an experience that turned out differently than you expected? What happened and how did you feel at the time?
5. Regarding this last experience where you wished things had turned out differently, did you or your doctor do anything to rectify the situation?
6. Did your participation in the study change your outlook on the way you manage your exacerbations?
7. What do you think would be the best way to integrate this type of long-term follow-up so that it addresses the aspects that are most important to you in the management of your exacerbations?
8. Is there anything else you wish to tell us about?

**Guide for the focus groups with care teams.**

1. From the point of view of the health care team, what are the most important aspects in the management of patients’ respiratory exacerbations, particularly in their daily lives?
2. In your opinion, how have the proposed monitoring methods, including connected devices and patient education, addressed these priorities or with what limitations?
3. During this research project, what changes have you noticed in the way the team works or in its workload with regard to monitoring patients for the management of their exacerbations? Have you noticed a change in your relationship with the patients’ out-of-hospital physiotherapist?
4. What difficulties or bad experiences have you had in the process of managing patient exacerbations using connected devices?
5. Do you feel that you had positive experiences during this study with the management of patient exacerbations? How would you rate these experiences in relation to the most important aspects of the management of respiratory exacerbations?
6. In your opinion, should this type of long-term patient follow-up be included in the management of exacerbations or in other aspects of their management? If so, what would be the best way to integrate it and for which patients and with which objectives?
7. Is there anything else you wish to tell us about?

### Analysis Framework

All the interviews were transcribed verbatim and subjected to a descriptive qualitative analysis. The analysis framework used was grounded theory [23].

Grounded theory is a qualitative research method with an inductive approach aimed at constructing a theory on a cultural, social, or psychological phenomenon by proceeding with the progressive and valid conceptual representation and mapping of qualitative empirical data [24]. In this study, the phenomenon explored was learning and empowerment in health management through the use of connected devices. Grounded theory is relevant as this phenomenon is currently sparsely studied. Studies on connected devices in patients with chronic conditions, and especially in patients with CF, are mostly intended to demonstrate the efficacy of the use of connected devices on various health outcomes. The theories mostly reported in the literature, such as digital behavior change interventions or the theory of reasoned action, are mainly focused on compliance with connected devices. However, the concept of empowerment includes other dimensions, such as understanding, the ability to decide, and self-assessment. Using grounded theory, we aimed to complete the current knowledge by eliciting the various dimensions of empowerment from the patient experiences with the use of connected devices for remote monitoring of their symptoms and by identifying elements that could enrich the theories in the field of remote monitoring.

According to the constructivist grounded theory method by Charmaz [25], which focuses on social processes or actions and the meaning of human actions, we adopted a social psychological approach to explore *how* and *in which context* individuals feel that connected devices have an impact on their learning to take care of themselves and on their empowerment.

In grounded theory, verbatim transcripts are analyzed using codes to highlight what was stated by the participants in the study and derive meaning from it. We applied the standard steps of grounded theorizing. In *initial coding*, we generated as many ideas as possible inductively from the initial data. In *focused coding*, we relied on the most prevalent and important codes to select the central codes for analysis. In *theoretical coding*, we refined the final categories of the theory by connecting them to each other, thus allowing for the integration of the categories into a model and the construction of a theory on the phenomenon studied [26].

The grounded dimensional analysis of patients’ or parents’ and health care providers’ data was conducted by 2 researchers (MM and DPB) using NVivo (QSR International) taking into account their evolution over the course of the study and the various natures and production conditions of the collected material while constantly comparing the data within and across patients or parents and health care providers. A constant comparison between the verbatim transcripts of patients and health care professionals was carried out to bring out the invariant elements as “the essence of the phenomenon,” which elaborate “conceptual categories” remaining as close as possible to the lived realities of patients [27]. The 2 researchers who analyzed the verbatim transcripts were a psychologist and the parent of a child with CF, and both had PhDs in public health and great experience in qualitative research.

### Analysis of the Educational Documents

The educational documents filled in by the physician and the patient during the educational session (educational workshop 2) were collected by the research team and reviewed globally but were not analyzed in connection with the patient interview. The aim was to understand which actions had been agreed upon between the patient and the physician when symptoms of a PEx were detected by the patient at home (central column) and whether they could resolve the PEx episode and prevent deterioration through their actions.

### Ethical Considerations

The research project was submitted for evaluation by the Committee for the Protection of Persons designated randomly under conditions provided for in the Code of Public Health (Article L. 1123-14). The study was approved by the Committee for the Protection of Persons (CPP North West III) on June 10, 2017 (2017-A00723-50). Free and informed consent was obtained before any act related to research was undertaken.

## Results

### Population Interviewed and Dropout Rates During the Study

A total of 56% (20/36) of the study participants were interviewed. The population interviewed in relation to the population included in the study and who benefited from the different stages of the educational program (educational workshops 1-3) is listed by center in Table 1. The dropout rate at the end of the first phase of intensive data collection (3 months) was 25% (9/36). In total, 3% (1/36) of the patients died during the study. The death was unrelated to the study. A total of 67% (24/36) of the patients were educated in the first 2 workshops (educational workshops 1 and 2), allowing them to enter the routine monitoring phase using connected devices. Only 39% (14/36) of the patients attended the third educational workshop held at the midpoint of the routine monitoring phase using connected devices. At the end of the study, the nonresponse rate to interview solicitations compared with the number of patients who entered the routine monitoring phase was 25% (6/24). These results differed from one center to another. The gender, age, and geographic area characteristics of the patients interviewed (presented in Table 2) were similar to those of the entire study population. However, the patients interviewed had a higher level of education and employment rate than the entire study population.

A total of 12 health care providers from 7 hospitals participated in focus groups between May 2020 and February 2021 (Table 3).

**Table 1:** Number of patients interviewed per center (n=20).

			Patients included (n=36), n (%)		Patients educated, n (%)							Patients interviewed (n=20), n (%)
					EW^a^ 1 (n=30)		EW 2 (n=24)		EW 3 (n=13)			
**Pediatric CF^b^ centers**												
	1	4 (11)		4 (13)		3 (12)		3 (23)		3 (15)		
	2	5 (14)		3 (10)		2 (8)		0 (0)		2 (10)		
	3	5 (14)		4 (13)		4 (17)		3 (23)		2 (10)		
**Adult CF centers**												
	4	8 (22)		6 (20)		4 (17)		3 (23)		3 (15)		
	5	6 (17)		6 (20)		6 (25)		3 (23)		5 (25)		
	6	3 (8)		3 (10)		3 (12)		1 (8)		2 (10)		
	7	5 (14)		4 (13)		2 (8)		0 (0)		3 (15)		

^a^EW: educational workshop.

^b^CF: cystic fibrosis.

**Table 2:** Characteristics of the study participants (patients; n=20).

Characteristic			Values
**Age**			
	Adolescents, n (%)	8 (40)	
	Adults, n (%)	12 (60)	
	Age of adolescents (years; n=8), median (SD)	14.5 (1.1)	
	Age of adults (years; n=12), median (SD)	29.6 (7.7)	
**Sex, n (%)**			
	Male	9 (45)	
	Female	11 (55)	
**Geographical area, n (%)**			
	Living in a city	12 (60)	
	Living near a city	5 (25)	
	Living in the countryside	3 (15)	

**Table 3:** Focus group participant characteristics.

Hospital	Participants (n=20), n (%)	Date	MD^a^ (n=3), n (%)	Nurse (n=4), n (%)	Physiotherapist (n=3), n (%)	Other (n=2), n (%)
1	2 (10)	November 6, 2020	1 (33)	1 (25)	0 (0)	0 (0)
2	3 (15)	February 5, 2021	1 (33)	1 (25)	1 (33)	0 (0)
3	3 (15)	November 9, 2020	1 (33)	1 (25)	1 (33)	0 (0)
4	3 (15)	October 11, 2020	0 (0)	2 (50)	1 (33)	0 (0)
5	4 (20)	December 9, 2020	1 (33)	1 (25)	1 (33)	1 (50; coach in physical activities)
6	3 (15)	June 23, 2020	1 (33)	1 (25)	1 (33)	0 (0)
7	2 (10)	May 19, 2020	1 (33)	0 (0)	0 (0)	1 (50; clinical research assistant)

^a^MD: doctor of medicine.

The forms completed by patients exiting the study reported technical difficulties with certain connected devices, in particular with the tablet computer provided to synchronize data before sending them to the research server. These difficulties were also reported by health care providers in the focus groups, who mentioned that a member of the team (nurse, physiotherapist, or clinical research associate) had spent a significant amount of time solving technical problems with the device suppliers, sometimes unsuccessfully. The reasons for dropping out of the study were multiple and are listed in Multimedia Appendix 3.

### Results From the Educational Documents

The educational documents collected after educational workshop 2 show that the first column (“normal state of health, routine activities and treatments actually followed”) was filled with detailed information on the treatments and activities of the patient in their daily life, unlike the central column, which contained little information. The agreed upon actions in case of signs of exacerbation were mainly “increase physiotherapy” or “try to do more physical activity” and always “call or send a message to the center team.” The actions were aimed more at the diagnosis of the exacerbation by the physician, who then decided what the patient should do, than at the actions that the patients should take by themselves. Most of the physicians added the following comment—“They already know what to do”—meaning that they had not delegated new actions to the patients. One pediatrician decided to give conditional prescriptions of oral antibiotics to the parents after the educational session, thus delegating to them the decision to start the treatment and asking them to inform the team that they had started the treatment.

### Descriptive Results From the Interviews

#### Stage 1: Initial Coding

A total of 12 codes emerged from the patients’ verbatim transcripts. In total, 10 codes emerged from the health care providers’ verbatim transcripts. The analysis allowed for the assignment of a name to each code that identified its area of interest (Table 4).

**Table 4:** Codes and categories of transcripts from patient interviews and focus groups with health care providers.

Category and codes from patient interview transcripts			Codes from transcripts of caregiver focus groups
**Category 1: patient empowerment**			
	Learnings New knowledge mentioned by patients that helps them understand alerts and manage PEx^a^	Learnings Confirmation of the patients’ perceptions of symptoms using measurements; better understanding of their state of health at the first signs of exacerbation	
	Empowerment Impression of being more capable of self-managing their treatments, their health, and their life projects	Patient-physician relationship Better understanding by the physician of the patient’s situation, their life circumstances, and their care; better understanding by the physician of the treatments carried out and the patient’s behavior in the event of exacerbation or in life in general	
	Loss of control Impression of being less capable of self-managing their care, health, and life projects	Remobilization of the team to manage PEx Renew the motivation of the teams to focus on the main objective of jointly managing PEx through a different approach with the patient	
**Category 2: TTF^b^**			
	Perceived usefulness Needs expressed by patients to monitor PEx and expectations of the use of CDs^c^ to help them self-monitor	Usefulness of monitoring using CDs Depending on the patient’s health status (unstable or stabilized), on the caregiver’s previous experience with telemonitoring, and on the patient’s ability to use devices and keep them in good operating condition	
	Perceived reliability Patients’ level of trust in the reliability of the data collected by the devices during the study	Technical reliability and accuracy of measurements Checking the accuracy of the measurements taken using CDs in comparison with hospital standards and reliability over time	
	Negative experiences Problems encountered using CDs; negative consequences described by patients	Negative experiences Problems encountered using CDs and negative consequences described by people—1 death that was not related to the study but that CDs did not prevent	
**Category 3: use of technology by patients and health care providers**			
	Conditions for a favorable use of CDs Technical, human, and environmental conditions of CD use considered favorable for the optimal management of PEx	Conditions of integration of the use of CDs into the organization of care Technical, human, and organizational conditions for the health care team to integrate the support of the use of CDs by patients—resources and time needed for education and remote support of patients	
	Motivation Personal and contextual factors that motivate patients to use CDs Hindrances Personal and contextual factors negatively affecting the use of CDs	Factors of motivation in health care providers Monitoring method that cannot be overlooked considering the current demographic increase in the number of adult patients; necessary monitoring method (using telecommunications) in case of a crisis (COVID-19)	
	Support from health care providers in the use of CDs Support provided by health care providers in the use of data from CDs for the management of PEx that helps promote the use of CDs by patients	N/A^d^	
	Use of CDs Modalities of CD use reported by patients	N/A	
**Category 4: recommendations and suggestions for long-term monitoring using CDs**			
	Patient recommendations Recommendations made by patients for an empowering use of CDs in managing their PEx	Implementation approach for the routine use of CDs in the organization of care The approach should define at which patients it is aimed, with possible adjustments over time; with which devices to collect which data (eg, when, how much, and the utility of alert notifications) with which devices to collect which data; which health care professionals will supervise this kind of routine monitoring; and the support from health care providers in the use of data from CDs to monitor patients.	

^a^PEx: pulmonary exacerbation.

^b^TTF: task technology fit.

^c^CD: connected device.

^d^N/A: not applicable.

#### Stage 2: Focused Coding

#### Overview

Following the analysis of the initial codes, the codes from patients’ and health care providers’ verbatim transcripts were used to construct unified categories (Table 4) fed by the diversity of patients’ and health care providers’ perspectives. These categories are independent from one another and do not include the same codes. At this stage of analysis, some codes (“Patient recommendations” and “Implementation approach”) were set aside as they did not correspond to the modeling purpose. They will be considered later in the model. Each category was defined with a general title, a description, and detailed transcripts.

#### Category 1: Patient Empowerment

During the process of analysis, empowerment was defined as individual empowerment, characterized by the learning achieved during the intervention, the decisions and actions implemented by the patient for their care or health, and their sense of control over their health. Connected devices allow patients to access data on their health status daily to monitor episodes of PEx, prevent them, and adjust the course of action when they happen. They contribute to making some patients more autonomous in the early management of PEx by supporting their decision-making and ability to take action without seeing or contacting primary health care providers:

CDs allow for a better assessment of one’s health status, and to take better care of oneself. It helps to be more autonomous and to avoid waiting until we are very sick to go to the doctor’s. It also helps to complement one’s care with extra physiotherapy, more sports, things like that...

Adult patient

The use of connected devices in the management of PEx not only allows for the adoption of preventive behaviors or better adherence to medical recommendations. Through their use of connected devices, patients also learn to manage their health with new data about themselves that confront the objective evolution of their health status with the way they feel and the effects of their lifestyle and their attitude toward their care. This process is characterized by the acquisition of new knowledge of one’s state of health, the validation of subjective perceptions, a better understanding of what happens on a physiological level, and focusing more attention on certain monitoring indicators. These learnings can be observed as early as during adolescence:

Well, I found that the fact that I could make my own measurements ... allowed me to understand better...to be able to compare and to feel when I was not doing so well. And for example, I found it interesting when I thought I was doing less well but still had good results. I waited a little while to see if I should rely more on the results or more on how I felt, and I actually relied more on my results. And, yes, I thought it was good, because it’s mostly meant for prevention, and it helped me a few times.

Teenage patient

However, it has been found that measurements reflecting a deterioration can lead to higher stress levels and a loss of empowerment when no action plan has been put in place in advance with health care providers. A lack of patient education to support the understanding of data, including the meaning of alerts, can cause a feeling of helplessness in patients, especially if the caregiver also appears to be confused by the new monitoring method. Obtaining data on one’s health status on a near-continuous basis only enhances patient empowerment if the patient possesses the skills to interpret and act on the data. Similarly, connected devices lead patients to think almost constantly about their health despite not always being in the right mental state to do so:

In the past, I probably used to desaturate without really realising it. I probably had headaches, but there you go...But now, I’m constantly stressed out, because I check my measurements pretty much all the time.

Adult patient

Well, no, I can’t see the results of the measurements on the connected devices, and when the nurse called customer services, she was told that it was me who had the data anyway. But I don’t understand the data, and neither does she. So, perhaps we need to be taught how to interpret them better or to get clearer explanations in the alerts we receive.

Adult patient

Health care providers focused more on the concordance between the perceptions reported by patients and the data collected than on patients learning to manage their PEx. Empowerment was seen by health care providers as patients’ ability to detect PEx and respond to it to limit its effects. Health care providers’ objective was to ensure the *effectiveness of the device* in improving patients’ state of health. Some health care providers emphasized the *beneficial relational change* fostered by the educational intervention that accompanied the implementation of connected devices, owing to which they communicated better with their patients. This allowed them to better understand their living conditions with the disease and how they cared for themselves daily. From their point of view, the data collected using connected devices increased patients’ level of information and of awareness of their condition. This gave the team the feeling of having a new tool to involve patients in the management of PEx and, thus, the capacity to influence the evolution of the disease:

Behind the word “anticipation,” we mean they should know how to spot the early signs and manage to put things in place and then call us. They should know not to wait for one or two weeks before calling to tell us they haven’t been feeling well for two weeks. So, for me, the study had an aspect of therapeutic education, thanks to the information panel (“React with CDs” Tool) that allowed us to sit down with the patients and have them think a little bit about what exactly they were doing.

Health care provider

#### Category 2: Adequacy of Technology (Combined With Education) to the Needs of Patients

Often found in the literature as “task technology fit” (TTF) [28], this category includes aspects related to the reliability of the devices, the accuracy of the data measured (in comparison with a standard), the ease of access to the data, and how adequate the educational program is, all of which shape patients’ perception of how well this “technology” fits their monitoring needs. Patients expressed concern that the devices should accurately reflect their condition. The adequacy of the devices for monitoring purposes can be assessed based on several criteria over time as patients experience the use of connected devices. The first criterion is the perceived reliability of connected devices over time and the accuracy of the measurements compared with measurements taken at the hospital:

When I took several measurements, I sometimes got very contradictory results. I sometimes wasn’t sure whether it was reliable.

Adult patient

The second criterion is the ergonomics and ease of use to connect to the tablet to access data and send them to the research server, which enables the sending of alert notifications:

But the fact is that the spirometer...it does not save the results. So, I could get a good score at the beginning, but I tried again and because I coughed a little bit the result wasn’t so good, so I started again from the start, but it’s a bit difficult. Results should be saved automatically.

Teenage patient

The third criterion is the technical support provided for the implementation of the devices:

What bothered me was that the curves on the graph—there were two curves—I never knew what they represented. And I even asked the nurse, and the nurse replied: “Indeed, it’s weird, what does it mean?” Even she searched for an explanation. To this day, I don’t actually have the answer...

Adult patient

These aspects were supposed to be controlled in the context of interventional research, but some patients had a disappointing experience even though they were aware that they were participating in a pilot study that would sometimes involve “teething problems”:

We told them that they were the first ones to go through all the steps and that everything was not necessarily perfectly set up for them...I told them that future patients would have fewer difficulties because we would manage to solve some things with them in the study. They tested the tools from beginning to the end and therefore experienced all the computer bugs.

Health care provider

The reliability of the devices used to monitor patients was mainly assessed by health care providers in comparison with the measurements taken using standard hospital equipment. This reliability was, from their point of view, guaranteed by the research context. Some patients felt that their health care providers did not have answers to the technical problems they faced:

I received several emails from the CF centre telling me that they were not getting the data. But I assured them that I was sending the results. I managed to show them that I had uploaded the data...I went onto HealthMate as I was getting an update every Sunday by email for the Withings devices. So, I forwarded it to them, and in fact, they said that the data were loading, but not on the research server.

Adult patient

Patients’ interest in technology may vary according to the connected devices proposed, the need they feel to monitor certain health indicators, and the attractiveness of the device. Moreover, patients may not wish to use them for fear of being confronted with poor results on certain critical measurements for the patient (or for the physician):

So, I found the sleep analysis option rather useful. Because I do sleep well at night, but I cough without realising it. I was either a little tired when I woke up in the morning, or even not at all tired, while it turned out that I had exacerbations at night. So, I could see that from two criteria: the first one was the decibel peak levels at night, and then the second one was when I didn’t have a restful night’s sleep. So, these were two rather useful criteria, I think. And then...yes, there also was a third one...It is my heart rate, which increased as soon as I coughed.

Adult patient

The integration of technology and patient education into the care process was seen as an additional workload by health care providers. Although dealing with technical problems took more time than expected for those in charge of the study (nurse or clinical research associate), physicians mainly mentioned the time spent on the patient education workshop (education workshop 2). Patient education undertaken by physicians in the adult patient care pathway is new for some adult centers, and those centers hope to benefit from a “return on investment” from it in the future. From the point of view of the care team, taking measurements using connected devices adds to the time already spent by patients managing their disease daily:

For us, it takes time, but obviously, for the patients it represents a lot of time too. In patients’ daily lives, it clearly adds minutes to their basic treatment. In terms of the team’s workload, it obviously adds work, and the therapeutic education workshops linked to the protocol were particularly cumbersome. It’s a lot of work at the time, but it clearly is really beneficial for the future.

Health care provider

Although the educational tool proposed in the study (educational workshop 2) was generally appreciated by adult patients, it may have seemed complicated to the adolescent audience although it was developed by a pediatric team and tested with several teenagers before releasing it to be used for research:

Therapeutic education went well too...The information pane (“React with CDs” tool) was really well done, and it allowed us to look into many habits that we didn’t have, well at least that I didn’t have.

Adult patient

The dashboard was not bad, but super complicated to use for a teenager. There is too much stuff on it. And clearly, too much information on the same page. You can’t go straight to what you’re looking for...I mean, you really need to look for it. In that sense, I think this table needs to be more legible, because there was a lot of data on it. And reading a lot of data in a table with many columns, it’s...it’s not appealing.

Parent

Personalized *alert thresholds* were set for each patient based on data collected during the first phase of the study following the statistical analysis (cumulative sum control chart). However, these alerts were rarely used by patients to manage their exacerbation episodes as reading measurement results alone allowed them to understand their health status or the lack of updates to thresholds rendered the alerts irrelevant:

At the end of the year, my FEV1 had increased by quite a bit, so when I started the new year with a new secondary infection, my FEV1 didn’t drop lower than the year before. As a result, I never received any alerts. So, I think in this case, we need to update the thresholds, because things can really fluctuate.

Adult patient

Questions emerged among health care providers on the profile or profiles of patients for whom it is more relevant to introduce self-monitoring measures via connected devices. The inclusion criteria of the study targeted patients with good to moderate lung function (FEV1 >50%) so as to limit the risk of patients leaving the study because of lung transplantation, which is considered as soon as FEV1 decreases to <40%. Some physicians who followed adult patients believed that stabilized patients are good candidates for this follow-up through connected devices, whereas others pointed out that very unstable patients could benefit from this reactive warning system to manage decompensation. In such a critical situation, physicians emphasized the importance of systematically transmitting patient data to the center to help monitor the patients using alerts. Although most physician investigators wanted the study not to send patient data to the center as they felt that they did not have the resources to treat them, other physicians considered it not to be viable for patients who were critically ill. The fear of widening existing social inequalities in health was also mentioned by the care teams:

I think it is useful to integrate the use of such devices with severely ill patients who have frequent exacerbations, who are hospitalised...It can really have a positive impact by confirming the patient’s perception that they are not doing so well, and that they may need to begin an intravenous treatment. It can help patients and us, health care providers, for patients who are severely ill, by providing objective data on exacerbations.... But at the same time, we must not delude ourselves. It is with these severely ill patients that it will be more difficult to set up a monitoring process with CDs. Because they often are in complicated situations socially, psychologically, and so on. So, I don’t know whether it will really be possible with these patients. There are biases and inequalities that will remain true with CDs. Whereas patients who are already autonomous and stabilised will more easily appropriate the CDs.

Health care provider

However, some patients want to maintain control over their data and make decisions themselves as they feared that connected device monitoring would increase the control of the care team:

I don’t need a doctor’s supervision to tell me to be careful and that today’s measurement was not good. Because on the contrary, I find it more worrying than anything else. But then, it depends on the CF centre. For example, some CF centres will use the measurements and overprescribe antibiotics, while others will want to see the patient in consultation.... It should be up to us, it’s our responsibility.

Adult patient

#### Category 3: Device Use by Patients (and Health Care Providers)

In the context of this study, device use refers to the ways in which patients used connected devices, whether continuously or intermittently, which may have evolved during the course of the intervention according to factors linked to the patients’ life circumstances, what they experienced during the study, and the conditions of integration of the new monitoring process into the organization of the care team’s work. These uses reflect patients’ perceptions of the *benefit-risk balance* of the technology and its evolution during the study. Patients adapted the frequency of their connected device use to their need to self-monitor between quarterly visits to the center or, instead, to their need to “let go” slightly on disease management. This need for monitoring increases in periods such as the introduction or cessation of treatment, and it fluctuates depending on life circumstances (work), events related to the environment (high pollen count), or symptoms linked to the disease.

The following is an example of patients’ need for self-monitoring in between consultations at the hospital:

This allows us to watch the evolution of our data. The problem is that we go to the hospital once a month, or even every three months. So, we don’t have a regular follow-up as such. Whereas with these devices, for example, if I do a spirometry test once a week, I get a score every week, and I will check quite regularly, either it is effective or it is not. It’s complementary to my usual care and it could perhaps help patients be more autonomous.

Adult patient

The following is an example of adopting connected device monitoring in specific situations or for particular diagnoses:

I am planning to get pregnant, and therefore, I think connected devices will be very useful during that time. Indeed, I may not be able to take all the treatments that I can usually take when I am not pregnant. So, I think the devices will be useful then and I also think I’ll be more conscientious in such circumstances.

Adult female patient

The use of connected devices also depends on the way measures are integrated into the patient’s personal organization, also known as the *routinization process*, which, when compatible with their lifestyle, can alleviate the feeling of burden related to the use of devices and contribute to making the collected data more reliable. In the absence of a routine, the use of connected devices can also be taught through therapeutic education sessions and become part of a *self-normative* approach connected to the patient’s perceptions of their health status:

I do it when I have a quiet moment before leaving in the morning, before physiotherapy, and that’s it. I always tried to do it in the same conditions, so that it wouldn’t skew the data.

Adult patient

In the particular case of adolescents monitored using connected devices, their use was *regulated* by the parents, which adds to the burden of preparation and control of certain treatments. The collaboration with an out-of-hospital physiotherapist in this monitoring was seen as a relief for the parent caregiver, and it emphasizes the importance (credibility) of the follow-up for the adolescent patient. The question of maturity related to patients’ age was raised regarding the implementation of monitoring using connected devices in adolescence. Conversely, a parent mentioned the help that these connected devices could bring for the *empowerment of young patients*. Additional notification functionalities inspired by other applications could also support their use of connected devices:

The greatest thing that could happen for kids would be that the watch sent them a notification if the scores were low and told them what to do. For example, we would set up some instructions onto the app, and as a result, they would receive notifications with the steps to follow on their watch. It would really make them autonomous then. Some apps allow the creation of a schedule and then send out notifications. Youngsters just have to look at their watch and it reminds them they have to bring a check on Monday at 10 AM to the school secretary to pay for the canteen. So, it doesn’t replace the parents, but it would relieve them of the task of always repeating things like a parrot, which causes a lot of conflicts in families.

Parent

Sometimes, connected devices reactivated conflicts between parents and adolescents regarding the fear of addiction to the tablet for uses other than health monitoring or because they give parents access to data on the adolescent’s behavior:

There’s a very intrusive aspect to it. It feels quite overbearing for teenagers to know that they have lost 200 grams and that mum and dad want them to eat more to get the weight back on. Parental monitoring of sleep also creates conflict, and it was the case for almost all teens, with the parents saying: “You’re going to bed too late, that’s why you’re tired, it’s not healthy for you...” Some parents decided not to look at the data for that reason.

Health care provider

The use of connected devices by patients is also determined by the interest and attention that health care teams pay to discussions on these data during consultations, phone calls, or teleconsultations with patients, which we will refer to as “patient support”:

So, I thought it was nice. It was really...I was sending screenshots of the saturation, well you know...and the FEV1. We had real conversations, and I found it interesting. It was more precise, less vague, the explanations I had to give...I had to give numbers, you know...

Parent

Conversely, when health care providers fail to take into account information from connected devices in patient monitoring, it can make patients *doubt* the importance and usefulness of such data, which, in addition to the burden of taking measurements, can lead to a lack of interest in these devices:

We talked about it, but then, we didn’t focus the consultations on it at all...I expected there would be more guidance in terms of therapeutic education...we did it once about the information panel (“React with CDs” Tool), it took a very long time, it lasted almost two and a half hours. But I expected it would be that way during consultations, precisely to teach us to manage it ourselves...Sometimes, I wonder how it would be like if we had a chat every month, just for five minutes, just to ask me if things were going well, if there were any problems, or if I thought something was wrong.

Adult patient

Beyond the use of connected devices for monitoring purposes between consultations, some patients suggested that this follow-up could allow them to *space out their visits* to the center, particularly when they lived far from the center or to limit the risk of contamination at the hospital:

Not on a regular basis, but sometimes when needed, to avoid going to the CF centre, because I live a little over an hour away. So, to avoid the journey, especially if I’m going to the hospital just to do a spirometry test to analyse FEV1, then yes, I might as well do it with the device at home...it allows me to do the measurements myself. At the time of the consultation, we can either have a video call or talk over the phone, and then, we just give the results...in addition, we can extract our data, so we can even send them by email and the doctor can look at them beforehand.

Adult patient

#### Stage 3: Theoretical Coding

**Figure 2:**
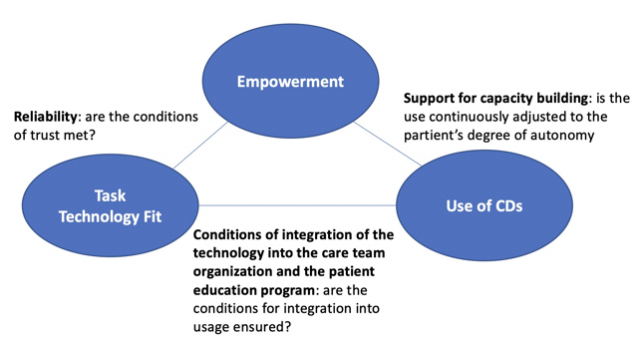
Category modeling and mapping. CD: connected device.

The comparison of verbatim transcripts in the 3 categories revealed the relationships between them, as shown in Figure 2. These bidirectional relationships can be explained as follows. First, the TTF–empowerment relationship: this is reflected in patients’ *trust* in technology as a necessary condition to consolidate their learnings, which in turn strengthens their trust in the support they receive from technology. Second, the empowerment-use relationship: patients’ capacity to take action and their feeling of control over their health condition with the way the technology is used, which in turn strengthens their capacity to act on their health. This relationship is *mediated* by the support provided by the care team to help patients adjust the use of technology for their daily management of the disease and, therefore, improve the PEx diagnoses and the *suitability* of the prescription. Third, the TF-use relationship: the adequacy of the technology to patients’ needs influences its use by patients, reflecting the perceived advantage for patients of being monitored using devices compared with their “standard” follow-up at the CF center. Patients use devices more if they seem adapted to their needs and if they are reliable and easy to use. This relationship is *mediated* by the care team’s *appropriation of the technology*, which translates to their coordination of the remote monitoring, the use of real-life data in patient education, and them learning to master the use of devices for patient care.

Connecting all the results leads to theorization, the final stage of grounded theory analysis, which can be formulated as follows. The use of connected devices by patients results in an increase in their ability to take action over their health (empowerment) through the continuous adjustment of this use to their degree of autonomy, which influences and is also influenced by the conditions in which the technology is integrated into the organization of the care teams and the patient educational program. The motivation of patients with CF to use connected devices to prevent and manage PEx is dynamic. It depends on the patients’ priorities and specific concerns as well as triggers that will increase the feeling of usefulness related to the connected devices. The data provided by connected devices become a source of new knowledge (eg, about their disease and health) and capacities (eg, to prevent and manage) if a learning process to use them in daily life is implemented. This learning process can be supported by patient education.

This theory accounts for the conditions under which the implementation of connected devices for the management of PEx in patients living with CF can increase their capacity to act on their health.

## Discussion

### Principal Findings

Our study theorized the conditions that favor individual empowerment in patients living with CF in the management of PEx using connected devices as part of the MucoExocet study. This study took place in the context of a rare disease, proposed by the health care team to patients who could be interested in the technology. This theorization of the individual empowerment of patients through the use of connected devices is provisional, similar to any theory derived from the grounded theory approach, and remains subject to verification.

Our study places the concept of individual empowerment through the use of technology at the center of our research. Indeed, empowerment is an important mechanism of eHealth self-management, but validated assessment tools are rare [29]. In our theorizing, we viewed empowerment as *the ability of individuals to identify and meet their own needs, solve their own problems, and mobilize the necessary resources through connected devices and education provided by health care providers to feel in control of the management of PEx* [10]. This leads us to consider the success of the implementation of connected devices from the perspective of the patient empowerment outcome, weighted by a typology of the intensity of connected device use by the patient deduced from our results. First, low use: patients doubt the feasibility and usefulness of this continuous monitoring; they are in favor of a “standard” follow-up at the CF center using data collected from the clinical examinations in situ. Second, advanced use: patients know that this monitoring can be useful in case symptoms appear or when a follow-up appointment at the CF center is not easily accessible. The use of connected devices remains optional and selective between the “standard” clinic visits at the center. Third, high use: patients experiment with the use of connected devices when in particular situations or because of particular behaviors, enabling them to consolidate or develop skills in relation to their health and the factors that influence it. This use is connected to their desire to improve their health and control its evolution and to the belief that the use of connected devices can support them in doing so. Depending on the patient’s situation (eg, developmental, emotional, and environmental), the level of motivation to engage in less or more intense connected device use will vary.

We suggest that empowerment, adherence to treatment, and quality of life be favored as primary outcomes of remote digital follow-up. In a recent study using a randomized trial that compared 2 groups of patients (using the tracker device *against* not using the device), Wildman et al [30] highlighted that the health improvement objectives were not achieved but that the intermediate objective of improving adherence to treatment was exceeded. These findings tend to confirm that, when assessing how effective the implementation of technology is with patients, the improvement of health indicators may not be the first outcome to be expected. This strengthens the case for a patient-based evidence evaluation approach.

In addition, the identification of “opposite cases” encountered in our study, for which patient empowerment was compromised, supports the theory stated—cases in which the devices were unreliable (TTF) or no action plan was defined in response to alerts or variations in measurements or cases of difficulties reaching health care providers (lack of support or difficulty integrating the use of technology into their organization) all led to lower levels of patient empowerment. Our study questions eHealth-backed education models, for which data are currently scarce. Following the work of Greenhalgh et al [31], our results highlight the interaction among the patient, the device, and the organizational and social system as the cornerstone of the learning process in patients. This interdependence underscores the systemic approach to connected device implementation, wherein connected device adoption and use and the positive experience with them cannot be attributed to the patient’s lack of motivation alone. Indeed, connected devices introduce a technopedagogical transformation among health care providers, which pushes them to rethink organizational and educational activities to support a new relationship with patients.

This study shows that connected devices may have enabled health care providers to gain a new understanding of patients thanks to the quality and novelty of the information obtained via connected devices. In this sense, connected devices could help bridge the gap that is sometimes observed between theoretical models based on medicine that is “centered on the person and their family” and the practice of care that lacks understanding of patients’ experiences in daily life [32]. Health care providers are made to understand the daily lives of patients living with a chronic disease in physical, psychological, and social terms, thereby creating a more symmetrical relationship of information sharing [33]. Our study shows that caregiver-patient interactions are modified by the introduction of connected devices. They are enriched by a new outlook on patients’ daily lives mediated by technology, which leads to a new understanding by health care providers.

In addition, this study confirmed that the implementation of connected devices should be considered based on patients’ health goals and not simply focused on education on the device [34]. Patient empowerment depends on the connected devices’ capacity to meet the needs of patients’ health project. Patients then enter a learning process supported by the connected device and with educational support from health care providers structured in 4 phases, as described by Almalki et al [35]: identification of an area of interest (the patient is focused on a specific health goal that requires the collection of data about themselves), personal analysis (analyzing one’s behavior in light of the objective data collected), self-experimentation (structuring a reasoning based on the trends identified in support of the experimentation carried out with the connected device), and activation (confirmation of the hypotheses made during the experimentation phase and development of personal knowledge). This process must be structured and accompanied to unfold properly.

Although a recent review of the literature [36] on the use of mobile devices (phones, patient monitoring devices, digital assistants, and other wireless devices) by patients with CF has shown medical, psychological, and behavioral benefits as well as benefits in terms of level of satisfaction with care, the psychological aspect has thus far received little attention, as is the case with the educational dimension of technological devices. In adolescence, although the disease significantly influences the development of one’s body image and self-concept [37], the integration of new technologies into self-care leads to a new understanding of oneself and, therefore, to a potentially modified relationship with one’s body, health, and illness. This process is an integral part of the use of new technologies. Therefore, it is a potential topic for future research, which is necessary to understand the use of new technologies in care and their effects on people.

### Limitations of the Study

This pilot study was based on an interventional research protocol. On the one hand, this protocol was implemented differently depending on the centers and the devices selected for the research—the teams applied the educational program differently, the elaboration of self-management action plans in the event of exacerbations was done differently (educational workshop 2), or the midterm review session of the routine follow-up of the patient was different (educational workshop 3). These differences in the implementation of the protocol were noted when collecting the experience of patients and data from the focus groups; they contributed to enriching the definitions of the categories and the relationships between them. In contrast, within the framework of this research protocol, we could not modify the tools that proved to be unreliable (which had been selected in 2016 via a market analysis while planning for the study), adjust the formatting of the data, or more generally adapt the intervention according to the results collected throughout the early phases of the study. Thus, having a protocol that is too fixed is probably a mistake to avoid in health technology research if we wish to adapt the intervention during its implementation to explore the best way of using health technology. In the context of the intervention, the choice of connected devices and the setup chosen did not allow patients to access a dashboard displaying the data collected by all connected devices. Some more motivated patients created their own dashboards separately. Furthermore, the pulmonologists in charge of patient follow-up were not always involved in the study, and this dichotomy made routine monitoring more complicated for patients. Similarly, when the clinical research associate in charge of the study was not the patient’s coordinating nurse, the latter was unable to answer patients’ questions during consultations or phone calls. Eventually, the study included people interested in technology, which could have biased the results based on the experience of using technology.

If this had been a descriptive pilot study, a quality improvement approach would have allowed for adjustments and improvements to the intervention over the course of the study and would have been directly driven by the care team. This format has been used to introduce connected devices into the patient care process for CF in the United States [38], which enabled patients to be equipped and monitored remotely when CF centers were closed during the COVID-19 pandemic by using a connected spirometer coupled with teleconsultation. The quality approach allowed for the evaluation of the results during the course of implementation, and adjustments were made to the intervention to improve its impact. The results were convincing:

In March 2020, the beginning of the pandemic, 37% (49/131) of patients owned a HS (home spirometer) and around 50% (9/20) of patients seen via telemedicine performed spirometry at home. By September 2020, 97% (127/131) of adult patients at UVA owned a HS, and by October 2020, 96% (24/25) of patients provided spirometry results during their telemedicine encounters.

### Prospects for Transferability

Assessing how transferable the theory could be outside the context of its development would require studying the introduction of connected devices in other circumstances: with patients living with different diseases, using different devices, or with a different organization of care.

Two opposite contexts could be studied in terms of patient empowerment through technology: (1) a context of patient dependence on self-regulated or caregiver-driven technology, whether it is telemonitoring, implantable devices for which the use is predetermined (dependence on technology and on health care providers making the care decision in the event of an alert or emergency), or protocolized treatment with little margin for adaptation or action because of side effects (eg, protocol dependence in cancer treatments); and (2) a context of patient-developed technologies [39] made available to patients living with the same condition in open source, as is the case with type 1 diabetes mellitus (T1DM). T1DM has the highest degree of patient empowerment and has recently led to the publication of an international consensus for the guidance of professionals caring for patients who use such devices. The case of T1DM is also interesting as research was conducted on the transition of patients from devices that allow for the management of glycemia and insulin delivery in a semiautomated way to a closed-loop insulin delivery system, which is designed to “free” the patient from self-management by automating the process of insulin delivery. However, this specific case might also lead patients to feel that they lose control over their glucose levels before they take back control over some other parameters of the automated process.

### Contribution to an Extended Theory of Empowerment From Remote Monitoring for Health Symptom Tracking

A recent publication by White et al [40] reports on a systematic review to help define engagement with remote monitoring for health symptom tracking (RMT) and how to measure it. Engagement is seen as a mediating factor that eventually explains the impact of RMT on patient health outcomes. Their analysis is of most interest to our own work and shows that concepts still need to be clarified in the context of RMT. They propose a definition of engagement through a remote monitoring protocol (dropouts), objective engagement, subjective engagement, and interactions between objective and subjective engagement. Although objective engagement (with remote monitoring itself, with symptom tracking compliance, and with app use of statistics) is clearly measurable, subjective engagement appears to gather a wide range of concepts, some of them from the technology acceptance model literature (usability, TTF, satisfaction with the technology, utility for symptom management, ease of use, and intention for future use; Davis [41] revised by Venkatesh et al [28] and Chang et al [42]).

In a further extended theory, we would rather build on certain determinants of the technology acceptance model and distinguish them from the concept of patient engagement. These determinants leading to the “behavioral intention of use” would be the *personal characteristics* (age and sex, expectations, social influence, hedonic motivations, and previous experiences with information and communication technologies), the *facilitating conditions over time*, *TTF* (over time as technologies are continuously refined), and *the mediating factors* (perceived ease of use and perceived usefulness). We would propose to include the “engagement with the research protocol” by White et al [40] as a determinant, renamed as “conditions for the RMT introduction/intervention” (either research or routine care or self-care). Our study aimed to add elements to modulate the “behavior use” in the RMT context, which is not explained by the previous theories and not necessarily consistent with the “behavioral intention of use.” From our study, these elements could refer to *patient empowerment*, such as their learnings about their own body, their trust in the technology, and the relationship and support they receive from their care team. We agree with the conclusion of White et al [40] to explore the RMT field in its own right as separate from Digital Behavior Change Interventions or general eHealth literature.

### Conclusions

Our study allowed us to propose a theory on individual patient empowerment through the use of connected devices based on patients’ and health care providers’ experiences in the context of an interventional pilot study. This theory needs to be validated with a larger sample and verified in the context of different diseases, different devices, and a different organization of care. It implies that, if the empowerment of patients with chronic diseases is indeed a desirable goal for all parties involved (patients, health care providers, and the health care system), the necessary conditions for the successful implementation of connected devices cannot be looked at separately for each party (health care providers, patients, and health care system). On the contrary, these conditions must be adjusted to the overall collaboration among these stakeholders, who cooperate toward patient empowerment. Only if all these conditions are met can patient empowerment be the outcome of the use of technology.

## Appendix

Multimedia Appendix 1 The “React to PEx” educational tool.

Multimedia Appendix 2 Exit interview questionnaire.

Multimedia Appendix 3 Reasons for leaving the study.

## Abbreviations

CF: cystic fibrosis
EQUATOR: Enhancing the Quality and Transparency of Health Research
FEV1: forced expiratory volume in 1 second
PEx: pulmonary exacerbation
RMT: remote monitoring for health symptom tracking
T1DM: type 1 diabetes mellitus
TTF: task technology fit
