# Supplementary figures and images for: Experiences Among Patients With Cystic Fibrosis in the MucoExocet Study of Using Connected Devices for the Management of Pulmonary Exacerbations: Grounded Theory Qualitative Research
Source: JMIR Form Res. 2024 Jan 23;8:e38064. doi: 10.2196/38064 (PMC10848132; doi:10.2196/38064)

**Appendix 1: The education tool « React to PEx »**

**
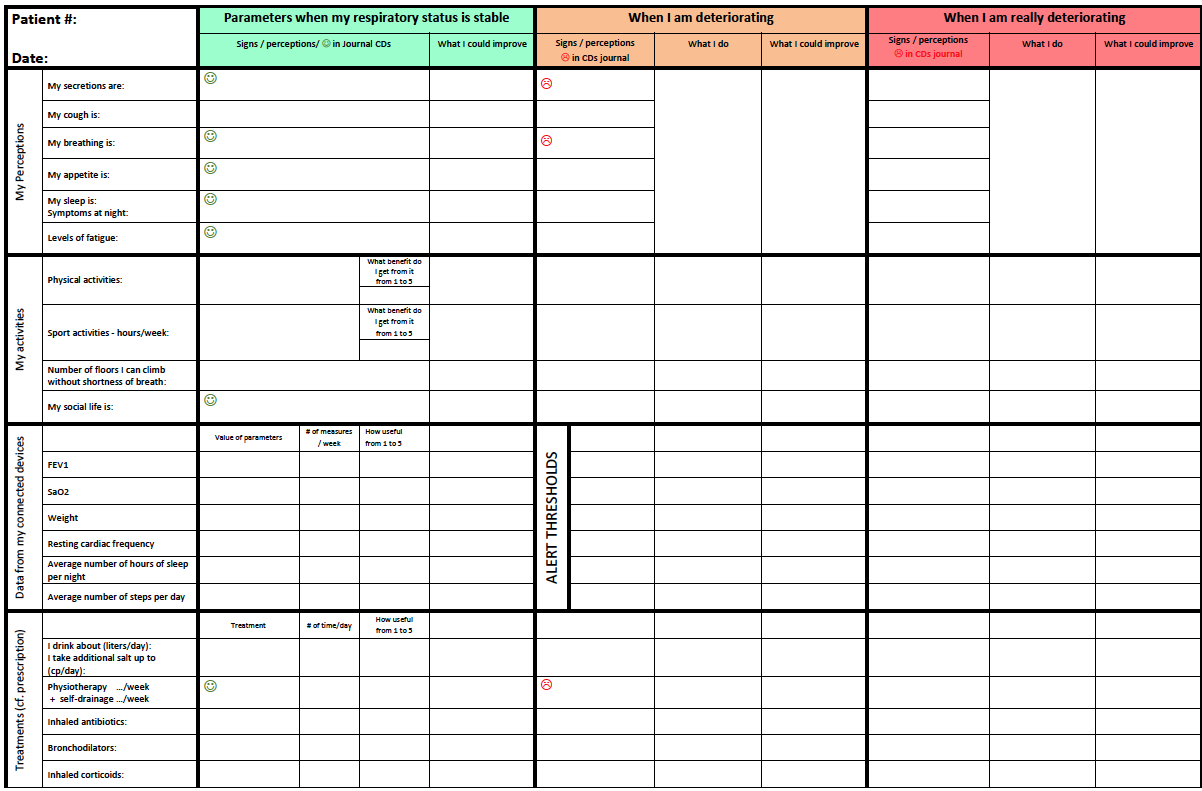
**

Supplement: Multimedia Appendix 1 [file formative_v8i1e38064_app1.docx]
