# Supplementary material for: Experiences Among Patients With Cystic Fibrosis in the MucoExocet Study of Using Connected Devices for the Management of Pulmonary Exacerbations: Grounded Theory Qualitative Research
Source: JMIR Form Res. 2024 Jan 23;8:e38064. doi: 10.2196/38064 (PMC10848132; doi:10.2196/38064)
Supplement: Multimedia Appendix 2 [file formative_v8i1e38064_app2.docx]

**Appendix 2: Exit interview questionnaire**

Thank you for taking the time to fill in this questionnaire. Your answers are strictly anonymous. They will be sent for analysis, under conditions that guarantee their confidentiality, to the people in charge of this research. Response time will not exceed 10 minutes.

**Patient Code: .../.../..**

1/ Evaluation of the use of connected devices for the early detection and treatment of pulmonary exacerbations in patients living with cystic fibrosis (MucoExocet)

Please tell us below why you refuse to participate (or refuse to allow your child to participate) in this study (free response):

………………………………………………………………………………………………………………………………………………………

………………………………………………………………………………………………………………………………………………………

………………………………………………………………………………………………………………………………………………………

2/ Please select the reasons why you would like to leave this study from the list below (multiple answers):

□ I feel uneasy about such connected devices;

□ I am concerned that my personal data will not remain confidential;

□ I lack space for these connected devices in my home;

□ I don't believe these connected devices are reliable;

□ I am afraid to break the connected devices;

□ Using these connected devices gives me a sense of intrusion into my life;

□ I have difficulties using the connected devices;

Specify: ................

□ I feel the monitoring by these connected devices is useless;

□ I lack time in my daily life to collect data from connected devices;

□ I couldn't collect data using connected devices daily for another reason;

Specify: .......

□ I am suspicious about the use of research results;

□ My current treatments are too burdensome;

□ I feel a sense of helplessness in the management of exacerbations;

□ My current management of exacerbations is effective;

□ I am not assiduous enough for this type of monitoring;

□ I prefer to be entirely monitored by the CF centre team or the out-of-hospital physiotherapist and without any self-monitoring of my data;

□ I am not affected by exacerbations;

□ Too complicated to manage in my daily life (shared custody...);

□ Other (please specify): ...............................................
