# Supplementary material for: Experiences Among Patients With Cystic Fibrosis in the MucoExocet Study of Using Connected Devices for the Management of Pulmonary Exacerbations: Grounded Theory Qualitative Research
Source: JMIR Form Res. 2024 Jan 23;8:e38064. doi: 10.2196/38064 (PMC10848132; doi:10.2196/38064)
Supplement: Multimedia Appendix 3 [file formative_v8i1e38064_app3.docx]

**Appendix 3: Reasons for leaving the study**

- **I feel the monitoring by these connected devices is useless = 4**
- **I lack time in my daily life to collect data from connected devices = 4**
- I forget to take measurements = 1
- My current treatments are too burdensome = 2
- My current management of exacerbations is effective = 2
- **I prefer to be entirely monitored by the CF centre team or the out-of-hospital
  physiotherapist = 3**
- I am anxious to see the results of my weight measurements = 2
- **The spirometry tests take too long because of connection problems or connection issues with the tablet = 5**
- **The CDs are too complicated to use = 3**
- I am afraid to break the devices = 1
- I am not assiduous enough for this type of monitoring = 2
- This type of monitoring is too complicated to manage in my daily life = 2 (teenager at boarding school, adult lorry driver)
- It gives me a sense of intrusion into my life = 1
